# Role of microbiome in autoimmune liver diseases

**DOI:** 10.1097/HEP.0000000000000506

**Published:** 2023-06-27

**Authors:** Kai Markus Schneider, Martin Kummen, Palak J. Trivedi, Johannes R. Hov

**Affiliations:** 1 Department of Medicine III, University Hospital RWTH Aachen, Aachen, Germany; 2 Norwegian PSC Research Center, Department of Transplantation Medicine, Oslo University Hospital Oslo, Norway; 3 Institute of Clinical Medicine, University of Oslo, Oslo, Norway; 4 Department of Oncology, Oslo University Hospital, Oslo, Norway; 5 National Institute for Health and Care Research Birmingham Biomedical Research Centre, Centre for Liver and Gastroenterology Research, University of Birmingham, UK; 6 Liver Unit, University Hospitals Birmingham Queen Elizabeth, Birmingham, UK; 7 Institute of Immunology and Immunotherapy, University of Birmingham, UK; 8 Institute of Applied Health Research, University of Birmingham, UK; 9 Research Institute of Internal Medicine, Oslo University Hospital, Rikshospitalet, Oslo, Norway; 10 Section of Gastroenterology, Department of Transplantation Medicine, Oslo University Hospital, Rikshospitalet, Oslo, Norway

## Abstract

The microbiome plays a crucial role in integrating environmental influences into host physiology, potentially linking it to autoimmune liver diseases, such as autoimmune hepatitis, primary biliary cholangitis, and primary sclerosing cholangitis. All autoimmune liver diseases are associated with reduced diversity of the gut microbiome and altered abundance of certain bacteria. However, the relationship between the microbiome and liver diseases is bidirectional and varies over the course of the disease. This makes it challenging to dissect whether such changes in the microbiome are initiating or driving factors in autoimmune liver diseases, secondary consequences of disease and/or pharmacological intervention, or alterations that modify the clinical course that patients experience. Potential mechanisms include the presence of pathobionts, disease-modifying microbial metabolites, and more nonspecific reduced gut barrier function, and it is highly likely that the effect of these change during the progression of the disease. Recurrent disease after liver transplantation is a major clinical challenge and a common denominator in these conditions, which could also represent a window to disease mechanisms of the gut-liver axis. Herein, we propose future research priorities, which should involve clinical trials, extensive molecular phenotyping at high resolution, and experimental studies in model systems. Overall, autoimmune liver diseases are characterized by an altered microbiome, and interventions targeting these changes hold promise for improving clinical care based on the emerging field of microbiota medicine.

## INTRODUCTION

The liver is a unique anatomical and immunological interface of host–environmental interactions, receiving antigen-enriched blood from the intestines while posing a firewall to avoid translocation of gut-derived pathogens into the systemic circulation. The liver's ability to promote immune tolerance is essential to prevent overexuberant inflammatory responses to nutrients or commensals entering via the portal vein.^1^,^2^ However, despite sophisticated immune privilege, loss of tolerance can be observed in autoimmune liver disease (AILD), which manifests as autoimmune hepatitis (AIH), primary biliary cholangitis (PBC), or primary sclerosing cholangitis (PSC). These diseases are thought to be caused by a complex dynamic between environmental risks and genetic predisposition, in which dysregulated mucosal immune responses are implicated in causing hepatobiliary injury.

Herein, we provide a contemporary overview of the evidence supporting the role of the gut microbiome in the pathogenesis and progression of AILD. In doing so, we debate the possibility of disease-triggering and/or disease-promoting microbiome signatures, overlapping pathological concepts, and nominate future areas of research priority in gut microbiome/AILD.

## THE GUT MICROBIOME

**FIGURE 1:**
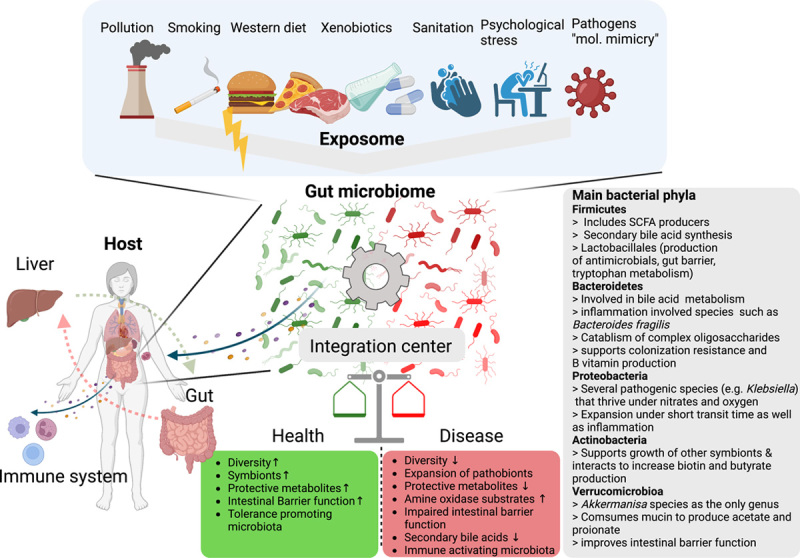
The microbiome integrates environmental influences into host physiology.

Body surfaces are colonized by an ecosystem of microorganisms consisting of bacteria, archaea, viruses, and fungi. The assemblance of all these microorganisms in a defined environment is called microbiota^3^,^4^ (Box 1). The entire habitat, including the totality of microorganisms in a living space, their genomes, and the surrounding environmental conditions, is called the microbiome. Bacteria make up large fractions of the gut microbiota (Figure 1), which compete in quantity with human cells and possess a number of genes far beyond the contents of the human genome.^5^,^6^ The gut microbiome functions as a metabolically active organ and switch point for the integration of environmental influences into host physiology,^7^ supporting important functions for digestion, maintenance of barrier function, mucosal immunity, and enzymatic reactions. Its plasticity allows rapid adaptation to changing environments, which might have served as an evolutionary advantage but comes with the prize of exposing some of the host’s vital metabolic machinery to environmental threats.^8^ These can be manifold, including Western diets, sanitation, reduced microbial diversity, excess alcohol consumption, and xenobiotic exposures (Figure 1).^9^

BOX 1 Important terms and definitions

Microbiome

Refers to the entire microbial environment. It includes not only living organisms, such as bacteria, archaea, eukaryotes, and viruses, but also their genetic material (ie, genes) and the surrounding physical and chemical conditions.

Microbiota

The collection of microorganisms existing within a defined environment.

Exposome

The exposome refers to the sum of all nongenetic exposures an individual experiences over the course of their lifetime, including environmental, occupational, and lifestyle factors.

16S rRNA gene amplicon sequencing

A sequencing technique used to survey bacterial (and archaeal) communities. Specific highly variable regions of the 16S ribosomal RNA (16S rRNA) are sequenced and then bioinformatically matched to a specific taxonomic group (e.g., a bacteria at a specific phylogenetic level), which can be used to analyze different aspects of the composition of a sample.

Shotgun metagenomics or metagenomics

Shotgun metagenomics is a genomic technique used to study a mixed microbial community by randomly sequencing DNA fragments from the entire genetic material in a sample. This allows the identification and characterization of different microorganisms (including bacteria, fungi, viruses, phages, and more) and their genes (functional potential) within the sample.

Metatranscriptomics

Metatranscriptomics is a genomic technique to study the entire set of RNA transcripts present in a complex microbial community, typically extracted directly from a sample. While metagenomics only studies the present genes and allows conclusions about the functional potential of a community, metatranscriptomics gives information about the expressed genes.

Beta diversity

Beta diversity is a measure of the variation in species composition between different habitats or communities. In clinical microbiome research, it is often used to quantify the degree of similarity or dissimilarity in species diversity and abundance between two or more individuals.

Alpha diversity

Alpha diversity is a measure of species diversity within a single community or habitat. It quantifies the number of different species (species richness) and the distribution of abundance across the species in a community (species evenness) within a sample.

Metabolomics

Metabolomics is a technology in biochemistry that involves the comprehensive analysis of small molecules, known as metabolites. Metabolomics studies can be performed on various biological systems, including cells, tissues, body fluids, and fecal samples. Metabolomics relies on analytical techniques, such as mass spectrometry and nuclear magnetic resonance spectroscopy, to measure and identify metabolites, and uses bioinformatics tools to interpret and integrate the data.

Various environmental influences (that we collectively term exposome) impact the gut microbiome, which acts as an integration center at the interface between the host and the environment and is centrally involved in a broad range of metabolic processes. To fulfill these functions, the human microbiome contains 5 main bacterial phyla shown in descending abundance.^10^ Unfavorable environmental exposures give rise to shifts in microbial composition and/or functions, which might promote, or even trigger, autoimmunity.

Collectively, environmental threats together with host factors may give rise to an unfavorable microbiota characterized by reduced diversity and expansion of pathobionts resulting in altered microbial metabolism, intestinal barrier impairment, and translocation of microbial components from the gut to the liver,^11^ which may shape the hepatic inflammatory microenvironment and regulate the balance between immune tolerance and activation. Altogether with its strategic position at the interface of the host and environment and manifold immunoregulatory functions, the microbiome could be one of the factors contributing to variability in the association between gut and liver phenotypes in AILD.

## OVERVIEW OF AUTOIMMUNE LIVER DISEASE

**FIGURE 2:**
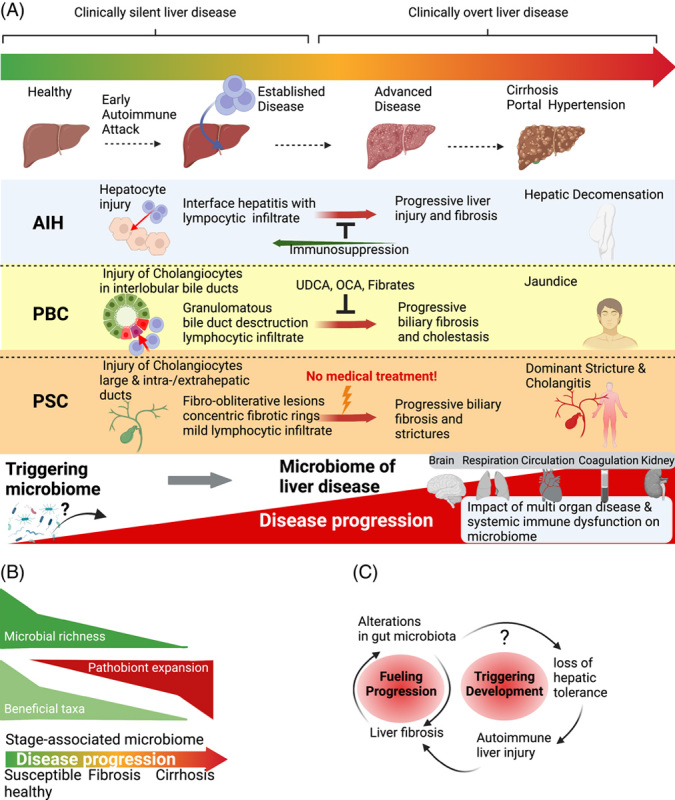
The microbiome during AILD progression. (A) Despite crucial differences in their pathophysiology, all autoimmune liver diseases share a disease course that progresses in different disease stages. In the early stages of the disease, AILDs are usually asymptomatic. At diagnosis, many already suffer from an advanced disease with potentially limited therapeutic options. Liver fibrosis and other organ dysfunction in advanced diseases also impact the microbiome. These confounders are blurring the disease-determining or potentially triggering microbiome signature. While disease progression can be slowed down in PBC or even (partially) reversed in AIH, PSC usually shows a progressive course with intermittent remission but with considerable variation in time from diagnosis to liver failure. (B) Disease progression gives rise to an unfavorable microbiome, associated with loss of microbial richness, expansion of pathobionts, and loss of beneficial taxa and their metabolic functions. (C) Alterations in microbiome composition and function might trigger autoimmunity but could, at the same time, be the result of advanced liver disease and fibrosis. Whether the microbiome serves as a trigger, promotes progression, or both remains unknown. Abbreviations: AIH, Autoimmune hepatitis; AILD, autoimmune liver disease; OCA, OCALIVA obeticholic acid; PBC, primary biliary cholangitis; PSC, primary sclerosing cholangitis; UDCA, ursodeoxycholic acid.

Broadly, AILD can be classified based on the target and distribution of autoimmune injury and further characteristics of the autoimmune response (Figure 2A, Table 1). While IgG4 disease and other systemic autoimmune conditions can also cause liver injury through autoimmune mechanisms, it is important to note that a comprehensive review of these conditions falls outside the scope of this work. The epidemiology of AILD differs in terms of both age and sex, with PBC and AIH predominantly affecting women, while PSC has a preponderance in men. Likewise, the absence of response to immunosuppression and strong association with concomitant inflammatory bowel disease separates PSC from PBC. Heritable aspects of autoimmunity are evident through family studies and specific monogenetic syndromes^12^–^14^ but hitherto identified genetic variants account for only 16% and 7.3% of the total heritability of PBC and PSC, respectively,^15^,^16^ highlighting a strong contribution from environmental factors or “the exposome” (Figure 1, Box 1). AIH is mainly a disease of hepatocyte injury,^17^ whereas, in PBC and PSC, immune-mediated damage affects the bile ducts. In addition, hepatobiliary injury in PBC is restricted to the interlobular bile ducts, while PSC also affects the large intrahepatic and extrahepatic biliary system.^18^

**TABLE 1:** Comparison of key characteristics between different AILDs

	AIH	PBC	PSC
Frequency	Incidence: 1–2/100,000/year Prevalence: 10–25/100,000^30^,^31^	Incidence: 1–2/100,000/year Prevalence: 22/100,000^32^	Incidence: 0.1–1.47/100,000/year Prevalence: 0–31.7/100,000/year^17^
Clinical presentation	Completely asymptomatic (12%–15% of cases), chronic liver disease, and acute/fulminant hepatic failure^33^	Asymptomatic, chronic liver disease, cirrhosis, and liver failure^34^	High fraction without symptoms at diagnosis, fluctuating disease progression ultimately leading to liver cirrhosis and failure^15^
Usual onset	Bimodal presentation: 10–20 years of age, 40–50 years of age^31^	> 50 years of age; Early onset – unfavorable prognosis^35^	30–40 years of age^36^,^37^
Sex distribution	AIH1: F:M = 4:1 AIH2: F:M = 10:1^38^,^39^	F:M = 1.6–10:1 Higher mortality in men^40^	M:F = 2:1^41^
Genetics/HLA	Early onset: *HLA-DR3* Late onset: *HLA-DR4*	*HLA-DRB1*	*HLA-B, HLA-DRB1*
Family history	Standardized incidence ratio 5.9^31^ No risk increase in second-degree relatives^31^	Familial history = strongest risk factor, sibling risk 10.5,^42^ Clustering within families^43^	Odds ratio in first-degree relatives 3.8–17.3^44^
Autoantibodies of clinical relevance	AIH1: ANA, Anti-SMA, anti-SLA, LP, AMA (rare – PBC overlap) AIH2: Anti-LKM1, SLC-1 Cave: Autoantibody negative AIH^38^	AMA, ANA, anti-SP100, anti-gp210, anti-lamin-B-receptor antibodies^45^	
Autoantibodies with putative gut-link	pANCA (anti-TBB5)	—	pANCA (anti-TBB5), anti-GP2
Clinical diagnosis tools	Autoantibody panels, IgG levels, and liver biopsy	ALP, Anti-AMA, liver biopsy	ALP, MRCP, Liver biopsy (small-duct/overlap)
Treatment	Prednisone, Azathioprine, and other immunosuppressive drugs	Ursodeoxycholic acid, obeticholic acid, bezafibrate	No medical treatment.
Prognosis	Untreated: <50% 10-year survival Treated: >90% 10-year survival^46^	77% LT-free survival at 10y^47^ Excellent following biochemical response to therapy	64%–94% 10-year survival (large variation between studies)^17^
IBD-association	Up to 16% UC^48^ *	Case reports	IBD in 20%–88% of cases^49^,^50^
Recurrence after liver transplantation	15%–40%^51^	14%–42%^52^	20%–25%^53^
Response to antibiotics	—	Rifampicin attenuates cholestatic pruritus^54^	Vancomycin, metronidazole, minocycline: lowered ALP values. Rifampicin: attenuates cholestatic pruritus.^55^

*Several of these with PSC-like cholangiogram.

The strong association between IBD and PSC (and, to a lesser extent, AIH) has led to several pathogenic hypotheses, wherein the gut microbiome has been proposed to contribute to, or even trigger, the development and progression of autoimmune liver disease. However, as the incidence of PSC is rising in western countries, that of IBD seems to have plateaued in Europe and North America.^19^–^21^ This contrasts with the increasing incidence rates of IBD in the East, wherein PSC is exceptionally rare.^22^ In any event, the immunology and characteristics of IBD in PSC could represent a window to studying disease mechanisms.^23^

In the next sections, we will cover in detail the clinical, epidemiological, and microbial evidence linking the gut microbiome to AILD. We will first deal with the concept of the microbiome as a trigger of autoimmunity in the liver. It is important to consider that the microbiome may influence disease and disease progression differently during the course of the disease. Microbial features that fuel disease progression might be different from those that trigger the development of autoimmunity (Figure 2B-C).

## CLINICAL AND EPIDEMIOLOGICAL EVIDENCE LINKING THE MICROBIOME TO AUTOIMMUNE LIVER DISEASE

Is the gut microbiome a trigger and/or a modifier of AILD (Figure 2A-C)? Individuals carrying genetic susceptibility may become exposed to triggers associated with a specific diet or societal urbanization in early life, altering their microbiota in a way that could trigger autoimmunity at a later timepoint. Importantly, gut colonization by microbiota occurs early in life,^33^ leading to the trafficking of microbial antigens to the thymus inducing the expansion of microbiota-specific T cells.^34^ Once in the periphery, microbiota-specific T cells have pathogenic potential or can protect against related pathogens. The mechanisms that elicit and balance inflammatory and tolerogenic responses are still being defined but may involve an early expansion of specific regulatory T cells following antigen exposures.^35^–^37^ While significant research has been conducted on the mechanisms of immune education and tolerance at the gut mucosal level, to date, there is a lack of experimental data to support the notion that the maturation of hepatic immunity is reliant on environmental cues provided by the gut microbiota during early life.

Data from the Dutch microbiome project indicate that only <7% of microbial taxa are heritable, whereas >45% can be explained by cohabitation,^38^ indicating that the microbiome is mainly shaped by environmental factors. More striking is the strong association between the adult microbiome and childhood environment, parental smoking history, and socioeconomic status. These discoveries provide insights into how differences in the gut microbiome may be shaped by varying exposomal factors in early life and correlate with temporal changes in the epidemiology of autoinflammatory diseases.^39^

Westernization of societies has been linked to the emergence of many immune-mediated diseases.^19^ In particular, the emergence of autoimmune diseases in low to middle-income countries is thought to arise from changes to microbial exposures, enteric dysbiosis, and food antigens.^39^–^42^ Epidemiological data show that the incidence of several autoimmune diseases has evolved over generations of migrant families to adopt that of the new country of residence.^43^,^44^ The identification of autoimmune disease “clusters” in specific geographical locations further supports an association between environmental risk and immune dysregulation.^20^,^45^–^47^ Liver disease–specific evidence is reported for primary biliary cholangitis (PBC), with greater incidence in urban postindustrialized versus rural areas in the UK.^45^ In Europe, a correlation between the incidence of AIH and PBC and the human developmental index has been reported, but no such association has been found for PSC.^48^ There is a lack of high-quality exposure data in AIH, but there is some evidence suggesting that antibiotic treatment one year before AIH diagnosis is an independent risk factor.^49^

The fundamental question of whether the microbiome predisposes to autoimmune responses has been addressed outside hepatology in a prospective study that studied microbiome signatures associated with the progression toward celiac disease (CD) in at-risk children.^50^ The authors identified several changes present 18 months before disease onset in children that developed CD compared to matched controls. The reported species have previously been linked to autoimmune and inflammatory conditions.^51^–^53^ Their presence before disease onset suggests that they may serve as biomarkers for future disease or triggers of autoimmunity. Unfortunately, similar studies are difficult to perform in AILD.

In experimental models, the translocation of the gut pathobiont *Enterococcus gallinarum* to the liver and other tissues was found to trigger autoimmune responses in mice, which is notable given that *E. gallinarum*-specific DNA can be detected in liver biopsies of people with AILD.^54^ Similarly, autoantibodies detected in individuals at risk of developing rheumatoid arthritis are seen to cross-react against gut bacteria in the *Lachnospiraceae* and *Ruminococcaceae* families. Specifically, the colonization of mice with a bacterial strain from the *Subdoligranulum* genus induced autoantibodies and eventually arthritis, akin to the pathology seen in human rheumatoid disease.^55^

Albeit being only correlative, a contribution of microbial triggers in PBC is highlighted by a higher risk of PBC in individuals with recurrent urinary tract infections.^56^ Similar data in PSC or AIH are less convincing but suggested by some.^57^ There are no consistent infectious factors linked to AIH or PSC, although there are several reports of chronic immune-mediated hepatitis following infection with endemic pathogens, including hepatitis A, Epstein-Barr Virus, and SARS-CoV-1, alongside mRNA- and adenoviral-based vaccinations.^58^,^59^ Moreover, the autoantigen detected by anti-Liver kidney microsomal type 1 (LKM1) is Cytochrome P450 2D6 (CYP2D6), which displays sequence homologies with human viral pathogens, such as HSV-1, HHV-8, and influenza A virus.^60^ Despite some clinical evidence and support from preclinical models, a specific microbial trigger for autoimmune liver diseases (AILDs) has yet to be identified. However, recent advances in sequencing and cultivation technologies offer the potential for unprecedented depth and scale in microbiome studies. In the next section, we will summarize recent clinical studies related to this topic.

## CLINICAL ASSOCIATIONS WITH MICROBIOME COMPOSITION IN AILD

A large number of studies have been performed in AILD since 2015, which are described in detail according to disease in Table 2. In the following, we will describe key patterns.

**TABLE 2:**
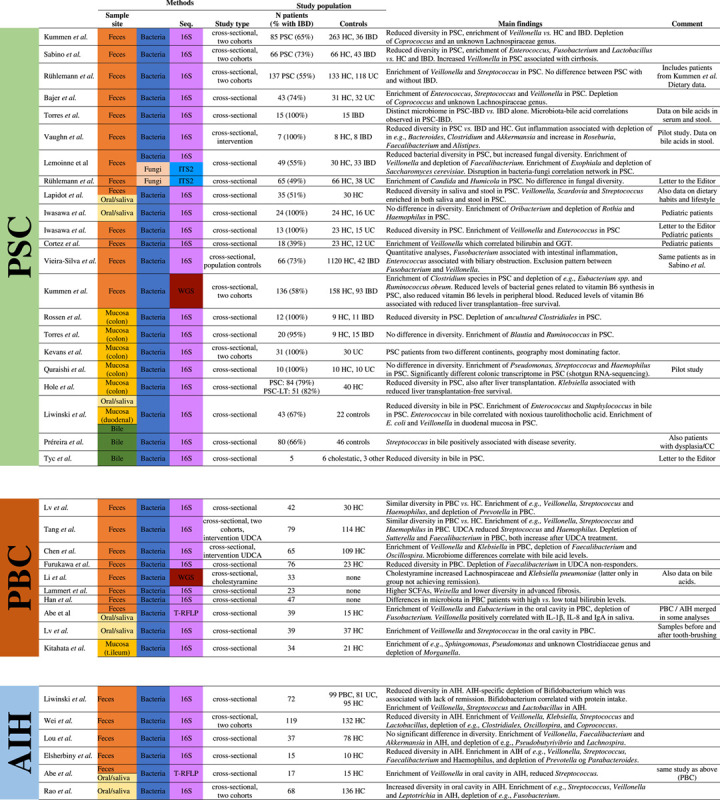
Microbiome studies in AILD

Abbreviations: 16S, 16S rRNA; CC, cholangiocarcinoma; HC, healthy controls; ITS, internal transcribed spacer; LT, liver transplantation; Seq, sequencing technique; t.ileum, terminal ileum; T-RFLP, terminal restriction fragment length polymorphism; WGS, whole genome sequencing.

### Reduced microbial diversity in AILD

The typical finding in AILD is reduced alpha diversity compared with healthy controls. With some exceptions,^61^,^62^ this has been reported in most studies of AIH and PSC.^63^–^70^ The data in PBC are more conflicting.^63^,^67^,^71^–^77^ Some of the few studies of mucosal microbiota in both PSC and PBC also showed signs of reduced diversity compared with controls.^78^,^79^ The only study directly comparing autoimmune liver diseases was by Liwinski et al, reporting on 72 individuals with AIH and large control groups diagnosed with PBC or UC.^63^ In this study, AIH and PBC showed similar alpha diversity at a lower level than healthy controls but higher than UC. Notably, this study also provided detailed data on dietary confounders, revealing no significant dietary differences in the intake of macronutrients or micronutrients between the groups after correction for gender and age.

Whether reduced diversity is caused by the liver disease itself or if treatment like antibiotics or ursodeoxycholic acid (UDCA) contributes is still not established. Reduced diversity could also reflect disease stage, as individuals with cirrhosis seem to have lower diversity compared to those without.^61^,^69^ While this has not been assessed properly, it is highly likely that the gut microbiota evolves during the course of liver disease (Figure 2A-B). Future studies of the gut microbiome in AILD should, therefore, assess disease stage and status in more detail. Another potential strong confounder is IBD in PSC, which may have an extensive impact on microbial diversity and metabolic functions.^80^–^83^ However, the large majority of microbiome studies in PSC report surprisingly little differences between individuals with and without IBD, but, usually, the activity of IBD is not assessed in detail.^68^–^70^,^84^

### Gut microbes altered in AILD

One often discussed hypothesis in microbiota studies is that individual microbes in a disease context may be beneficial or detrimental (often denoted “pathobionts”). While this highlights the possibility that specific bacteria could have pathogenic features, this view does not capture the full complexity of microbiota variation in disease, which can also be downstream of disease, a result of treatment, or just a confounding factor or an epiphenomenon. There are large variations between studies of reported differentiating genera (Table 2), and these changes have also been reviewed in detail.^85^ Some genera have been consistently enriched or depleted in several AILD studies and will be highlighted here.

A majority of studies on the fecal microbiota in PSC,^68^–^70^,^84^,^86^ including the pediatric populations,^87^–^89^ and also studies of the mucosal microbiota of duodenum and colon report an increased abundance of *Veillonella*,^63^,^79^,^87^,^89^ a group of gram-negative anaerobic cocci. Increased *Veillonella* has also been reported in PBC,^71^–^73^ AIH,^63^–^66^ in people with IgG4-related sclerosing cholangitis,^90^ and in liver cirrhosis of multiple etiologies^91^,^92^ and also nonhepatic conditions, for example, idiopathic pulmonary fibrosis,^93^ systemic sclerosis,^94^ and ileal Crohn’s disease.^95^ Only weak correlations have been observed between *Veillonella* and disease state, that is, with ALT levels in AIH 63 and with the Mayo risk score in PSC.^68^ The *Veillonella* often co-occurs with *Streptococcus*,^96^,^97^ which is also increased in all AILD,^62^,^69^,^84^,^86^,^71^,^72^,^74^,^63^,^64^,^66^ a co-occurrence that could partially be explained by their potential for metabolic interaction.^97^ It has also been shown that inflammation-associated nitrate in the intestine could be serving as a substrate and niche for *Veillonella*.^98^ Taken together, these data suggest that *Veilonella* thrives and expands in a gut environment influenced by liver disease, in particular, in advanced liver disease, while a role as disease cause or modifier has not been established.

Multiple microbes are depleted in the stool of people with AILD. Reduced levels of the *Lachnospiraceae* family have been reported in all AILD,^62^,^65^,^68^,^74^ and the depletion of *Lachnospiraceae* also amplifies hepatobiliary disease in experimental mouse models of PSC.^100^ In PBC, *Lachnospiraceae* increased following cholestyramine treatment and correlated inversely with bilirubin levels,^75^ but if or how these changes contribute to any treatment effect is not known. A marked depletion of *Bifidobacterium* has been reported in AIH compared to both healthy individuals and people with PBC, which is associated with failure to achieve complete biochemical remission.^63^ *Faecalibacterium* is also depleted in all AILDs.^63^,^72^–^74^ Finally, in the largest study of the mucosal microbiota in PSC, depletion of *Akkermansia* was found in PSC-IBD compared with PSC alone, representing the perhaps first overlapping features of IBD with and without PSC.^79^

The majority of the studies have used 16S rRNA sequencing for the characterization of bacteria, which cannot reliably identify bacteria at the species level. It is likely that we have to identify bacteria at the strain level and reveal bacterial functions if we are to detect their true role in disease, whether it is symbiotic or pathogenic. Studies applying whole genome “shotgun” sequencing can overcome some of these hurdles.^101^ These studies are scarce in AILD, but the first important publications are now available in both PBC and PSC (Table 2).

### Oral and biliary microbiome

The oral microbiome has also been studied in AILD. Low diversity does not seem to be a prominent feature in the oral cavity, but *Veillonella* and *Streptococcus* are reported to be enriched in PSC.^86^,^88^ The biliary microbiota in PSC has been characterized in multiple studies.^102^–^105^ Working with bile is challenging, in part because it generally has low microbial biomass, and PCR-based methods are sensitive to contamination from, for example, microbial remnants in lab solutions. Sterile sampling and negative controls at all steps are crucial, allowing bioinformatic “decontamination”.^106^,^107^ Still, some key observations have been made, and the biliary microbiota is dominated by common gram-positive bacteria, such as *Enterococcus* and *Streptococcus*, but also typical gram-negative pathogens within the Proteobacteria phylum, including *Klebsiella*.^102^,^103^,^105^

One important question is the cause and effect of microbes in the biliary tract. In a study of bile cultures from 189 people with PSC, a strong negative association was observed between positive biliary cultures for *Enterococcus* (present in 28%) and liver decompensation, transplantation, or death.^108^ *Enterococcus* is of interest as it is typically found enriched in the gut in PSC.^62^,^69^,^84^,^86^,^87^,^89^ Negative effects on outcome were also observed when bile cultures were positive for fungi,^108^ in line with previous associations between *Candida* in bile and poor prognosis.^109^,^110^ The presence of biliary antigens to mucosal-associated invariant T cells and natural killer T cells, which could be of microbial origin,^104^,^111^ is of interest since these atypical T cells could be speculated to modulate the bile duct inflammation, and mucosal-associated invariant T cells are enriched within the bile ducts in PSC.^112^ The question is whether this is colonization due to multiple infections and multiple endoscopic retrograde cholangiographys or whether the microbes of the bile ducts are driving progressive inflammation and stricturing. However, one study showed that the similarities between the microbiota of bile and the oral and duodenal microbiota were limited.^103^

Overall, the bile ducts are more often colonized in PSC, and the biliary microbiota is variable, often comprises pathogens, and associates with severity measures. Another important topic is the relationship to the development of CCA, which could potentially predispose to or be influenced by specific biliary microbes.^113^,^114^ Regarding AIH and PBC, little is known about the biliary microbiota, mainly due to the challenges of obtaining bile since endoscopic retrograde cholangiography is generally not indicated.

### Tissue microbiome

Finally, a topic that has emerged in recent years, although still controversial, is the presence of a specific microbiota within liver tissue. Recent studies have revealed that the liver is not a sterile organ, but rather, it may harbor a unique microbiome that can modulate liver immunity.^115^ Given the low biomass in these studies, strict contamination controls are essential. Studies specifically assessing the intrahepatic microbiomes in AILDs are eagerly awaited.

### The mycobiome and virome in AILD

Little is known about gut mycobiome and virome in AILD. In one study from France, the fungal composition was investigated in stool samples of 49 people with PSC, finding higher fungal diversity compared with IBD, and a trend toward higher fungal diversity than in healthy.^70^ People with PSC had enriched *Exophiala* genus and Sordariomycetes class, and were depleted in S*. cerevisiae* species. They were also able to show that patients with PSC have a disturbed fungal-bacterial network compared to healthy controls, a disturbance not observed in IBD. A letter published in connection with this study confirms an enrichment of Sordariomycetes in German individuals with PSC and also found an increase in *Candida* species.^116^

The recent advances in microbiology and sequencing technologies have provided an unparalleled understanding of microbiota composition and function in individuals with AILDs. However, these data are largely associative and partly inconsistent, many studies lack the sufficient size to make robust observations of parameters with large variability influenced by numerous unknown confounders, and few studies take into account the different stages of liver disease (Figure 2A).^117^ The next section, therefore, covers how the microbiota may trigger or shape the course of the disease and which methodologies that have been or should be applied to define disease mechanisms and improve clinical practice.

## PATHOGENETIC CONCEPTS AND EXPERIMENTAL EVIDENCE

**FIGURE 3:**
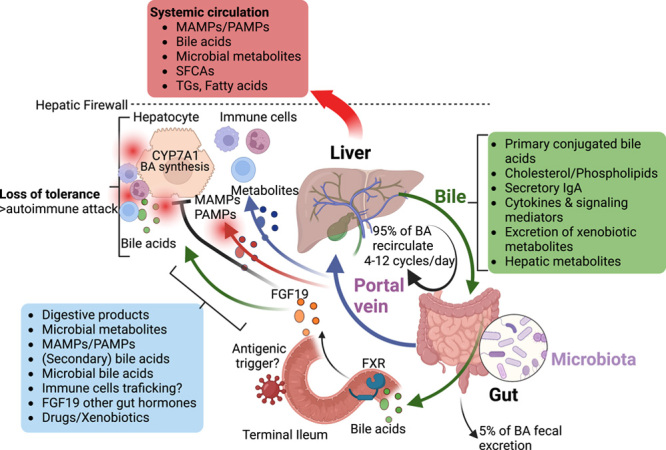
Mechanisms of gut-liver crosstalk in autoimmune liver disease.

The unique ability of the liver to promote immune tolerance is essential to prevent overexuberant immune responses to nutrients or microbial molecules entering through the portal vein.^118^ Certain pathogens can exploit the liver’s tolerogenic properties leading to chronic infection, whereas others can lead to severe inflammation, hepatocyte necrosis, or biliary fibrosis. Indeed, microbial triggers have been proposed as instigators of immune-mediated liver injury,^119^–^121^ fostering a temporal loss of tolerance to self-antigens. Unfortunately, delays in clinical presentation and/or diagnosis make identifying specific disease triggers difficult since these may be long gone at the time of diagnosis (Figure 2A). We also have learned from infectious diseases that only subsets of bacterial strains possess the virulence factors crucial for pathogenicity,^122^ increasing the need of knowledge at higher resolution. Furthermore, it is worth noting that, despite the small intestine being responsible for the majority of nutrient assimilation and absorption, most microbiota studies in AILD do not focus on this site. The small intestine plays a critical role in the complex interactions between microbiota and the host, owing to its significant contact with food substrates and its involvement in bile acid metabolism. As such, it represents a crucial area for future investigation in microbiota studies related to AILD.^123^ Microbiome research in AILD, therefore, relies on the combination of translational clinical studies and experimental models to dissect how complex changes in microbial community structure and metabolism affect host physiology (Figure 3). Of particular relevance in autoimmunity are factors that may initiate or perpetuate inflammation, which is why we focus on the role of gut barrier function, microbial drives of inflammation and immunity, and products of microbial metabolism that may modify inflammation.

The gut microbiome strongly engages in reciprocal gut-liver interaction. Bile released into the duodenum contains a broad range of molecules with signaling properties, such as primary conjugated bile acids, cholesterol, phospholipids, and cytokines. These molecules shape and modulate intestinal microbiome composition and function. At the same time, these molecules are subject to microbial metabolism, which critically shapes their signaling properties. Conversely, the portal blood is highly enriched in microbial metabolites, microbe-associated molecular patterns/pathogen-associated molecular patterns, and secondary bile acids, which influence parenchymal and nonparenchymal cells in the liver. A healthy liver has a remarkable potential to provide tolerance again harmless antigens while eliminating pathogens. However, intestinal dysbiosis may fuel hepatic inflammation and loss of hepatic tolerance. Whether certain microbes may directly trigger autoimmunity and under which context remains to be clarified.

### Gut barrier function

Bacterial antigens may cross the gastrointestinal barrier and translocate from the gut into the liver. Immune and parenchymal cells of the liver are equipped with pathogen recognition receptors, such as toll-like receptors or NOD-like receptors, which may trigger innate and adaptive immune responses. Intestinal barrier impairment and translocation of bacterial components to the liver have been implicated in various liver diseases but also autoimmune diseases outside the gastrointestinal tract.^124^–^127^ It is unclear whether barrier impairment is a cause or consequence of liver disease or autoimmunity.^128^ In mouse models, an unfavorable microbiota composition can lead to intestinal barrier impairment. Conversely, treatments with beneficial commensal strains, such as *Akkermansia muciniphila* or *Faecalibacterium prausnitzii*, have been shown to improve gut barrier function.^129^,^130^

Dedicated attempts to measure gastrointestinal permeability have been made in 2 studies in PBC, which both find increased sucrose excretion in a mixed sugar test, suggesting increased gastric and (proximal) small bowel permeability.^131^,^132^ There were no correlations with clinical markers or histological stage,^131^ indicating that barrier impairment might be a triggering event for disease onset but not sufficient to drive progression. In PSC, there was no evidence of reduced gut barrier function, as measured by the lactulose to l-rhamnose test.^133^ However, as there is some uncertainty about this methodology, these data should be interpreted with caution. Moreover, these studies did not examine intestinal microbiota composition, and it remains unknown whether gut barrier impairment is dependent on microbiota in PBC.

Circulating bacterial products or inflammation signals may serve as markers of gut barrier function. Early data showed an abnormal biliary accumulation of LPS in biopsies from people with PSC and PBC compared to controls suffering from obstructive jaundice, hepatitis C cirrhosis, or no disease.^134^ LPS and its interaction with toll-like receptors4 through CD14 are the prototypical examples of conserved bacterial components, which can directly activate pathogen recognition receptors and induce immune responses.^135^ In PSC, LPS-binding protein and soluble CD14 have been found elevated and predictive of poor prognosis, but not LPS itself.^136^,^137^ In PBC, elevated LPS and sCD14 have been found, and the latter is associated with poor outcomes.^138^,^139^ In AIH, circulating levels of LPS have been found elevated in a relatively small study of treatment naïve individuals. The investigators also observed reduced expression of tight junction proteins, which was associated with a lower abundance of *E. coli* and *Enterococcus*.^140^ Collectively, these studies suggest that the gut barrier function may be altered in AILD, but do these changes play a functional role in disease pathogenesis?

The altered gut barrier function is also seen in mouse models of biliary disease. Mice lacking the canalicular phospholipid transporter Mdr2 (*Abcb4*) develop spontaneous cholestasis and biliary fibrosis and serve as a valuable model to study disease pathophysiological mechanisms of PSC. *Mdr2* ^*-/-*^ mice develop an unfavorable microbiota composition, which has been linked to intestinal barrier impairment and increased translocation of LPS into the portal vein.^141^–^143^ This may trigger the activation of an NLRP3-inflammasome, which is a NOD-like receptor mediating an immune response and infiltration of inflammatory monocytes.^142^ Similarly, mild biliary inflammation was induced with dextran sodium sulfate in mice lacking only 1 MDR allele.^144^ In another study of *Mdr2* ^*-/-*^ mice, an increased abundance of *Lactobacillus gasseri* was found, triggering the expansion of IL17A-positive T-cell populations.^141^ Interestingly, the transfer of *Mdr2* ^*-/-*^ microbiota was sufficient to induce barrier impairment and liver injury in WT recipients, which provides strong evidence for the involvement of microbiota in the disease phenotype in mice.^142^

### Microbiota-mediated immune activation

The total mucosal surface area of the gut is about 32 square meters and harbors the largest collection of immune cells in the human body, which is continuously exposed to the external environment.^145^ The gut promotes tolerance toward harmless diet and microbiota-derived antigens and facilitates digestion and absorption of dietary nutrients across the intestinal epithelium and, at the same time, fights pathogens.^146^ With its high plasticity, the gut microbiota can be regarded as an evolving interface of host–environmental interactions. Its close interactions with the immune system allow the microbiome to shape innate and adaptive immune responses in the gut and beyond.^147^,^148^ Multiple cytokines have been found to increase in PBC, and the levels could be linked with changes in microbiota composition.^71^

Cross-reactivity between endogenous and microbial antigens has been proposed as an explanation for several autoantibodies in AILD, including perinuclear antineutrophil cytoplasmic antibodies (pANCA), which have been reported in up to 94% of people with PSC and up to 81% of people with AIH.^149^ It has been proposed that the target of perinuclear antineutrophil cytoplasmic antibodies is the nuclear protein tubulin beta 5, and there is a cross-reaction with the *E. Coli* protein FtsZ, an evolutionary precursor of beta tubulins,^150^ indicating that AILD might be triggered by an abnormal immune response to the intestinal microbiome. Furthermore, the presence of gut microbes was necessary for the development of perinuclear antineutrophil cytoplasmic antibodies and anti-FtsZ in a genetic mouse model of colitis. The results are compelling but have not been independently reproduced.^151^ Furthermore, no clear immunogenetic determinant of ANCA has been found in PSC, but the presence of ANCA was associated with a higher frequency of the strongest genetic risk factors.^152^ There was no difference in individuals with and without IBD, and overall, the clinical utility seems limited. This is also the situation in AIH,^153^ questioning the relevance of antineutrophil antibodies as disease drivers.

Similar theories have been put forward regarding antimitochondrial antibodies (AMAs) in PBC, related to urinary tract infections often caused by *E. coli.* 154 There are molecular similarities between human targets of AMA and *E. coli* protein, suggesting that cross-reactivity could induce AMA. Another proposed target is *Novosphingobium aromaticivorans*,^155^ which was present in the stool of 25% of people with PBC.

Stronger clinical associations are observed with IgA antibodies against glycoprotein 2 (anti-GP2), which are present in 31%–52% of the investigated individuals with PSC,^157^–^159^ but in very few individuals with PBC or AIH.^157^,^158^ Glycoprotein 2 is produced in the exocrine pancreas and released into the intestine, and it is also expressed on M cells in the intestinal barrier, where it binds fimbriated bacteria to mediate transcytosis.^160^ These bacteria-binding properties suggest involvement in immune responses toward the gut microbiota. Anti-GP2 is associated with more severe PSC and a higher risk of cholangiocarcinoma,^157^–^159^ in addition to sporadic cholangiocarcinoma and secondary sclerosing cholangitis.^157^ Anti-GP2 may, therefore, be a feature of biliary diseases, which is not specific to one condition.

An experimental link between microbiota, the gut barrier, immune activation, and AILD was described by Nakamoto and colleagues in a study where microbiota samples from people with PSC-IBD, and controls were transferred into germ-free mice.^161^ Such “humanized” mice engrafted with human microbiota are a valuable model system in functional microbiome studies. Pore-forming *Klebsiella pneumoniae* was isolated from mesenteric lymph nodes of mice inoculated with PSC microbiota, which caused intestinal barrier dysfunction resulting in more severe disease in mice with 3,5-diethoxycarbonyl-1,4 dihydrocollidine (DDC)-induced sclerosing cholangitis. This phenotype was likely driven by T-helper-17 (T_H_17) cell activation and could be rescued by simultaneous treatment with an RORyT inverse agonist, which selectively inhibits T_H_17 differentiation. Notably, the effect depended on additional bacteria, *Enterococcus gallinarum*, and *Proteus mirabilis*. The identified pathobionts were not exclusively present in PSC but also in some individuals with AIH or PBC. Data from microbiota profiling studies suggest an overall low prevalence of *Klebsiella* in PSC,^96^ but its presence in intestinal mucosa may be associated with reduced liver transplantation-free survival in PSC,^79^ and *Klebsiella* has been reported as increased also in AIH and PBC.^64^,^65^,^72^,^73^ The proposed mechanism could, therefore, be speculated to trigger T_H_17 responses also in these conditions, which aligns with human studies supporting the role of T_H_17 responses in AILD.^162^–^165^ T_H_17 cells are CD4 positive T cells that develop in mucosal surfaces like the intestine as a response to commensal microbes and are important in the protection against infections. However, T_H_17 cells have been implicated in many autoimmune conditions.^166^ In PSC, an increased T_H_17 response to microbes is seen, and monocytes from people with PSC are sensitized to produce higher levels of IL-1β and IL-6 known to drive T_H_17 cell differentiation upon stimulation with *Enterococcus faecalis* and *Candida albicans*.^167^,^168^

Taken together, animal models provide evidence that specific microbes may trigger autoimmunity.^54^,^55^ Although such data still remain correlative in human AILD, it is conceivable that pathobionts may be triggering autoimmunity also in human disease. A particular emphasis should, therefore, be put on identifying key disease-associated microbes with adequate specificity.

### Microbial bile acid metabolism

Bile acids are synthesized in the liver from cholesterol and are transported across the canalicular membrane of hepatocytes as primary conjugated bile acids into the biliary system, which finally drains them into the duodenum.^169^ Bile acids shape intestinal microbiota composition but are also subject to metabolism by gut microbiota.^170^ The majority (about 95%) of bile acids are reabsorbed in the terminal ileum and undergo enterohepatic circulation.^171^ In cholestatic liver disease, it has been postulated that an initial immune-mediated injury of the biliary tree (“first hit”) leads to obstruction of bile flow, accumulation of bile salts, and inflammatory response (“second hit”).^172^ Bile acids will also accumulate during the progression of the disease to cirrhosis in all AILD, but the bile acid homeostasis and composition may also be altered earlier due to changes in the gut microbiome, with unknown effects on the liver or other organs.

Importantly, microbial bile acid transformations alter their receptor binding properties and can modulate downstream signaling.^170^ The nuclear bile acid receptor farnesoid x receptor (FXR) is expressed in the terminal ileum and liver and acts as a major negative regulator of hepatic bile acid synthesis. Activation of ileal FXR induces the release of FGF19 into the portal circulation, which suppresses the rate-limiting enzyme of hepatic bile acid synthesis CYP7A1. The relevance of this is demonstrated by antibiotic treatment of *Mdr2* ^*-/-*^ mice, which impaired microbial conversion of the FXR antagonistic primary conjugated bile acid Tauro-β-MCA (not seen in humans), blocking FXR and abrogating negative feedback suppression of CYP7A1 resulting in severe cholestatic liver injury.^174^ A similar mechanism may also explain why germ-free *Mdr2* ^*-/-*^ mice have a more severe disease than conventionally raised mice.^175^ These data also show us that microbiota may have positive or negative effects on biliary disease depending on the mechanisms (*Mdr2* ^*-/-*^ versus *NOD.c3c4* described above) and illustrate limitations of murine models for studies of bile acid homeostasis in human diseases.^176^

May microbial bile acid metabolism modulate the activity of the FXR/FGF19/FXR axis in people with cholestatic liver diseases as well? Bile acid composition is changed in PSC^177^,^178^ and PBC.^179^,^180^ People with advanced disease often display suppressed BA synthesis, evidenced by the surrogate marker C4, which is associated with poor transplantation-free survival. It is unlikely that endogenous microbiota-mediated FXR modulation would improve disease outcomes since bile acid synthesis in these individuals is already fully suppressed.^174^,^178^ Therefore, it will be important to study the relevance of the microbiota-FXR axis in early autoimmune cholestatic liver disease. Beyond its effect on bile acid synthesis, enhanced FXR activation serves beneficial functions in AILD by improving intestinal barrier function.^181^

Specific bile acid species may also directly regulate adaptive immunity by modulating the balance of T_H_17 and T_reg_ cells.^182^ The derivates of the secondary BA lithocholic acid (LCA), 3-oxoLCA, inhibited the differentiation of T_H_17 cells by directly binding to the key transcription factor retinoid–related orphan receptor-γt (RORγt), and isoalloLCA increased the differentiation of T_reg_ cells. Recently, the human gut bacteria and corresponding enzymatic pathways converting the secondary bile acid LCA into 3-oxoLCA and isolithocholic acid were discovered.^183^ Strikingly, people with IBD displayed reduced levels of these bile acids and reduced expression of their biosynthetic genes, which correlated with the expression of T_H_17-cell-associated genes.^183^ IsoalloLCA may also reduce the risk of infection with gram-positive pathogens due to intrinsic antimicrobial properties.^184^ Data on the role of these secondary bile acid species in AILD are still missing. Bile acid homeostasis is likely to have a role in AILD beyond the FXR-FGF19 axis, and studying novel secondary bile acids and their impact on immunity in AILD is an important priority.

### Microbial metabolites beyond bile acids

The gut microbiome represents a biochemical factory, and microbiota-derived metabolites are mediators of host-microbial interactions. DNA-based studies of the microbiota provide a glimpse into the functional profile of these communities (Table 2). However, these data have to be analyzed with caution since metagenomics provides information about the genomic potential of the microbial community and abundance of genes, and not the expression of these genes or the level of metabolites actually produced, which is only possible using metatranscriptomics or metabolomics.^185^,^186^ In some conditions, by-products of microbial metabolism may influence the disease process and, hence, potentially represent clinical biomarkers or treatment opportunities. A prominent example is trimethylamine N-oxide (TMAO), which was discovered in cardiovascular disease.^187^ TMAO is generated in the liver from trimethylamine, which is produced by gut bacteria from precursors such as choline and carnitine, representing an interesting link between environmental (dietary) factors and disease.

Similar data on gut microbial metabolism in autoimmune liver disease are limited. In 1 study, TMAO was lower in PSC compared with controls, but, in a subset with normal synthesis, high levels are associated with reduced liver transplantation-free survival.^190^ The precursor of TMAO is trimethylamine, and amines are of interest in the context of studies of the vascular adhesion protein-1, relevant for T cell homing. T cells primarily activated in the gut express integrin α4β7 and may be recruited to tissues through interaction with the mucosal adhesion molecule MAdCAM-1, which may be expressed in PSC livers and also other diseases.^29^,^30^ T cell recruitment through this system requires vascular adhesion protein-1, which, in PSC, was found to be increased both in the liver and circulation 1.^191^ The activity of vascular adhesion protein-1 is substrate-dependent, and bacterial monoamines, such as cysteamine, are the most potent substrates. Cysteamine may also induce colonic cancer and colitis in mice, which makes these observations particularly interesting for our understanding of the disease of the gut-liver axis in PSC.^192^ Interestingly, a recent study has revealed that the commensal microbiota of patients with inflammatory bowel disease produces genotoxic metabolites that can cause DNA damage, potentially leading to colorectal cancer. Similar mechanisms may also contribute to the significantly increased cancer risk observed in individuals with PSC.^193^

Microbiome profiling in PSC has suggested alterations in the metabolism of essential nutrients, including the reduced potential to synthesize Vitamin B6 and branched-chain amino acids.^96^ Reduced concentrations of these metabolites in the blood are associated with reduced liver transplantation-free survival. These observations could point to a microbiome-driven susceptibility to vitamin B6 deficiency, although such a link is not proven. Little is known about vitamin B6 in PBC or AIH, except for a deficiency observed in advanced chronic liver disease.^194^ This could suggest that vitamin B6 deficiency is not a PSC-specific phenomenon but could be relevant for other AILDs.

Bacterial metabolites, such as SCFA, can activate specific receptors on immune cells.^195^ While SCFAs are generally considered beneficial, data on their role in AILD are far from clear. Some studies have reported a reduced abundance of butyrate-producing bacteria in PBC and AIH.^67^,^74^ Another study found higher levels of PBC, which were associated with SCFA-producing microbiome and liver fibrosis.^76^ Awoniyi and colleagues observed increased liver injury in *Mdr2* ^*-/-*^ mice treated with antibiotics.^100^ Vancomycin selectively decreased *Lachnospiraceae* and short-chain fatty acids (SCFAs) but expanded *Enterococcus* and *Enterobacteriaceae*. Furthermore, the authors observed increased hepatic translocation of *Enterococcus faecalis* and *Escherichia coli*. Interestingly, *Lachnospiraceae* colonization in the gut reduced liver fibrosis and the translocation of pathobionts in antibiotic-pretreated *Mdr2* ^*-/-*^ mice. Conversely, colonization of GF mice with *E. faeclis* and *E. coli* strains accelerated hepatobiliary inflammation and mortality. This study points toward the protective functions of the SFCA-producing microbiome in the *Mdr2* ^*-/-*^ mouse model of PSC. Whether SCFA is protective or not in human PSC needs to be clarified in future studies.

## EVIDENCE FROM CLINICAL INTERVENTIONS TARGETING THE MICROBIOME

### Pharmacomicrobiomics of drugs treating autoimmune liver diseases

Studies aiming to modify the gut microbiome to treat disease are critical to prove in humans that the microbiome is important in the disease process. However, an interesting first question is whether established therapies in these conditions target the microbiome and, in part, act through gut microbes. In population studies, strong associations have been observed between the gut microbiome composition and common drugs, such as statins and proton pump inhibitors.^196^ Interventional studies in humans and experimental models support such observations, and for some drugs, the pharmacomicrobiomic features may be part of the effect, for example, for metformin, which induces microbiome changes that by itself may improve blood sugar control.^197^

Regarding PSC and UDCA, which is commonly prescribed in PSC,^198^ the gut microbiome composition is generally similar in individuals with and without this drug.^68^ Notably, in a study of PBC, microbiome investigation before and 6 months after initiation of UDCA led to a normalization of 6 bacteria initially associated with PBC,^72^ which could either be part of the treatment mechanism or secondary to improved biliary function.

The IBD in PSC is commonly treated with 5-ASA drugs. There is some evidence that this influences gut microbiome composition,^199^ but there is so far no evidence that 5-ASA influences liver disease. It is also likely that immunosuppressants, such as glucocorticoids and azathioprine, may influence gut microbiome composition, but there is limited data from human studies^200^,^201^

### Antibiotic trials

Trials of antibiotics are interesting for 2 reasons. First, antibiotics might serve as effective drugs in AILD. Second, they serve as a tool to establish a causal link between gut microbes and disease. Agents such as minocycline and metronidazole reduce alkaline phosphatase in PSC.^202^–^204^ Several publications with vancomycin are observational and open-label or case series in PSC, some even in recurrent PSC.^205^–^208^ In adult PSC, 2 randomized controlled trials of vancomycin over 12 weeks have been performed, both suggesting that oral vancomycin reduces alkaline phosphatase levels, but there is no long-term follow-up data. In PSC in children, where the use of vancomycin was initially tested,^209^ a large retrospective study from an international consortium did not show improved survival compared with UDCA or no treatment.^210^ Overall, this leaves many questions unanswered, and there is a strong rationale for larger randomized controlled trials of oral vancomycin in PSC.

Most of the antibiotic trials did not perform deep molecular phenotyping like microbiome sampling before and after treatment. However, it is evident that vancomycin in the gut will remove the majority of gram-positive bacteria with subsequent loss of secondary bile acids,^211^ which depend on a biochemical machinery only present in a limited number of gram-positive bacteria. An outgrowth of gram-negative bacteria like Proteobacteria is also expected. Considering PSC-associated microbes specifically, *Veillonella* is usually not considered vancomycin sensitive. However, both *Streptococcus*, which is a key provider of lactate to *Veillonella*, and *Enterococcus*, which correlated with alkaline phosphatase levels in a quantitative study,^83^ are typical targets. The systemic absorption of oral vancomycin is minimal, but it has immunomodulatory properties, and whether these are mediated through antimicrobial actions is not known.^212^ Vancomycin has also been reported to be effective as therapy for IBD in PSC.^213^–^215^ This strengthens the rationale to study the effects of vancomycin on the PSC-IBD disease spectrum, but it will be critical to try to separate the effects of IBD from those on PSC.

In comparison to PSC, the experiences with antibiotics to treat AIH or PBC are limited. In an Italian retrospective series, vancomycin was given to 12 children with AIH or autoimmune sclerosing cholangitis with suboptimal response to immunosuppressive drugs,^216^ and 8 went into remission. However, data on antibiotics in AILDs other than PSC remain limited, and it is too early to make any conclusions about a potential therapeutic role in these diseases.

### Fecal microbiota transplantation

Like antibiotics, FMT represents a nonspecific intervention causing general alterations of the gut microbiota. FMT is tested in multiple conditions within and outside the gastrointestinal tract, both to define a therapeutic potential and as a tool to understand the link between the gut microbiome and disease. This is of particular relevance for PSC since multiple studies in ulcerative colitis (primarily without PSC) suggest that FMT may induce remission in a subgroup.^217^–^220^ The methods in these studies are variable, ranging from duodenal infusions to enemas and capsules, with variable numbers of donors. In PSC, an open-label pilot, including 10 people, has been published,^221^ where all received a single FMT during colonoscopy. The intervention appeared safe in the PSC population, increasing microbiota diversity and some fluctuations in alkaline phosphatase. However, the sample size was too small to conclude any signs of effect on the liver. Additional studies are planned in PSC, while only some murine^222^ but no human data are available in AIH or PBC.

### Prebiotics, probiotics, and bacteriophages

While FMT aims to replace the entire microbial ecosystem, its modulation is also possible through the supplementation of specific dietary components (prebiotics), individual bacterial species (probiotics), or combination preparations of prebiotics and probiotics, referred to as synbiotics.^223^ Probiotics are hardly studied in AILD. Only 1 study has been performed, in PSC, where 14 participants where administered a mixture of 4 Lactobacilli and 2 Bifidobacterium over 3 months in a cross-over study, with no clinical or biochemical signs of effect.^224^ Despite already being used as an over-the-counter medication, there are many fundamental questions that need to be clarified before use in a clinical context. For example, is colonization with probiotic bacteria required for the desired effect or is the effect mediated purely through bacterial components? It is also not known what conditions must be met by the host for colonization to occur. The molecular mechanisms of action are incompletely understood for most prebiotics and probiotics, and there is even data that they may do harm in certain conditions.^225^ Hence, prebiotics, probiotics, and synbiotics should probably only be used within clinical trials in AILD.

Finally, recent data highlight bacteriophages as potential tools to precisely edit the intestinal microbiome. This treatment has also been proven to be successful in murine experiments in the context of alcohol-associated hepatitis and PSC,^223^,^226^ and based on preclinical data, eradication of *Veillonella or Klebsiella* might be a potential target in PSC.

Altogether, several treatment modalities targeting the microbiome are on the horizon. The first results seem promising; however, most current therapies lack a personalized approach. Future studies should include deep longitudinal phenotyping with precision targeting of microbiota alterations.

## GUT MICROBIAL CONTRIBUTION TO RECURRENT DISEASE: A POTENTIAL MODEL FOR STUDYING AUTOIMMUNE LIVER DISEASES

A particular aspect of autoimmune liver disease is the risk of disease recurrence after liver transplantation. The diagnosis may be complex due to similarities to posttransplant complications, for example, ischemia or rejection; it is generally accepted that disease recurrence regularly occurs in all autoimmune liver diseases. The literature suggests incidences of 20%–25% in PSC,^227^ 15%–40% in AIH,^228^ and 14%–42% of PBC cases.^229^

Recurrence of AILD after transplantation is intriguing because it happens despite treatment with potent immunosuppression. Notably, autoimmune disease may also recur in other solid organ transplants.^230^,^231^ This suggests that disease mechanisms may not be solely immune-related, but rather, they may also be mediated by microbial metabolites. Alterations in gut microbial composition could be a possible contributing factor to disease recurrence. This hypothesis has a particularly strong basis in PSC, where the presence of IBD is one of the few risk factors repeatedly observed in studies of recurrent PSC,^232^ and pretransplant colectomy may protect the new liver from recurrent disease.^233^

**FIGURE 4:**
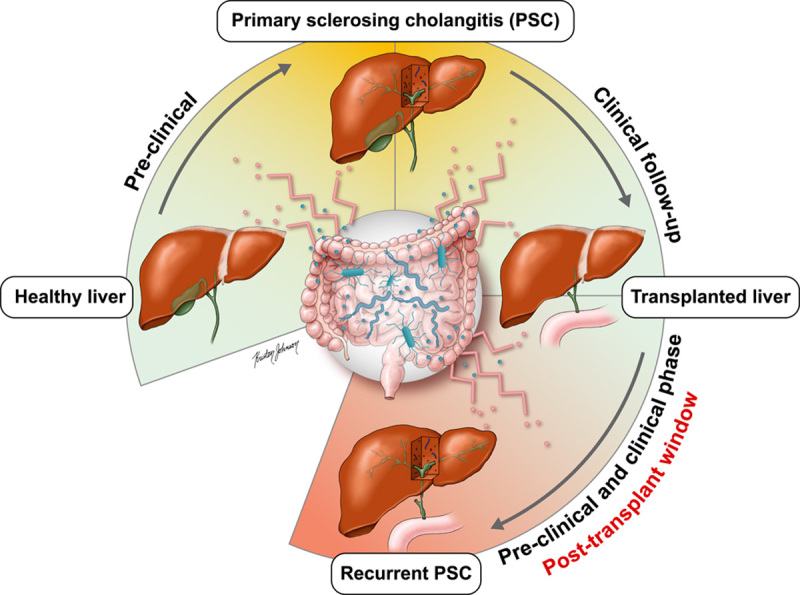
Natural history of an AILD before and after liver transplantation. PSC is used as an example, but both AIH and PBC are relevant parallels: Outline of the natural history of PSC (clockwise circle). There is probably a long preclinical phase from which we have scarce scientific documentation, followed by PSC diagnosis, transplantation, and recurrence in 20%–25%^227^ after liver transplantation. The figure illustrates how research on recurrent disease (a “human model”) in the posttransplant window could potentially shed light on disease mechanisms, which are active also before transplantation, for example, microbial factors from the gut. Abbreviations: PBC, primary biliary cholangitis; PSC, primary sclerosing cholangitis.

A persistent gut microbial factor driving recurrent disease would imply that the gut microbiome does not normalize after transplantation. This was recently observed in a Norwegian study of the ileocolonic mucosal microbiota of 84 persons with PSC and 51 persons who were liver-transplanted due to PSC.^79^ In the study, there was no sign of normalization of the microbiota composition after liver transplantation; rather, there was an expansion of, for example, potential pathogens within the Proteobacteria phylum. There were also some bacterial taxa overlapping between individuals with PSC before and after transplantation (ie, recurrence). Furthermore, in a study of fecal samples from recipients of liver or kidney transplants,^234^ persistent low-diversity dysbiosis was found for many years after transplantation and was associated with mortality. Taken together, the gut microbiota may influence posttransplant liver health in general and could also specifically be a disease driver in autoimmune liver disease. This could be used in future research by assuming that gut microbial factors and disease mechanisms are persistent in autoimmune liver disease both in the native liver and in the graft (Figure 4).

## CONCLUSIONS AND FUTURE DIRECTIONS

Genomic and metabolomics technologies have opened a new window to the pathophysiological mechanisms of AILD. The literature convincingly shows that the gut microbiome composition is different in individuals with and without AILD, but whether the changes observed are causing disease or are caused by disease is not known. Experimental evidence from mice suggests that individual pathobionts may trigger autoimmunity in susceptible individuals, but there is so far no direct clinical evidence of this in AILD. Little progress has, therefore, been made toward defining the triggering events or proving that these are microbial. However, the clinical observations following colectomy or treatment with certain antibiotics, as well as the worsening of experimental PSC following humanization experiments, all suggest that the microbiome very likely has a role as a disease modifier. New therapeutic targets may, therefore, be identified without having a complete understanding of the triggering events.

There are many tasks ahead to increase our understanding of the pathogenesis and invent new preventive and therapeutic alternatives. Among the major shortcomings and priorities are further descriptive work on humans to define disease-related microbes at a more detailed level. There is a need for better-characterized cohorts to reduce confounding from, for example, disease stage, IBD, and IBD activity, and provide data at higher resolution. These studies should also include individuals with control diseases and cirrhosis of other etiology to allow differentiation between the AILD microbiome and general alterations in liver disease. There is also little data on the biochemistry of microbes; there is a strong rationale for metabolomic studies of blood and stool to identify potential disease-driving microbial factors.

Many experimental tools are now well-established, with germ-free and gnotobiotic animals being used in experiments with the transfer of human microbiome, or colonization with a single bacterium or communities of limited complexity. Using these in conjunction with AILD models will be essential to prove cause-effect and also to study the actual disease mechanisms.

Experiments are also done in humans during clinical intervention trials. All trials should investigate the microbial domain to understand how treatment changes the microbiome. Furthermore, there is a need to define interventions that target the microbiome, including new diets. One of the mysteries in AILD is its variable and unpredictable disease course. Longitudinal data on microbiome trajectories during the natural disease course may eventually facilitate the development of better predictive models and uncover therapeutic opportunities. While the impact of microbiome research on clinical care in AILD has been very small so far, the overall activity and momentum in the field and discoveries in other conditions give hope of significant progress.

## Supplementary Material

SUPPLEMENTARY MATERIAL
