# Supplementary material for: Role of microbiome in autoimmune liver diseases
Source: Hepatology. 2023 Jun 27;80(4):965–87. doi: 10.1097/HEP.0000000000000506 (PMC11407779; doi:10.1097/HEP.0000000000000506)
Supplement: SUPPLEMENTARY MATERIAL [file hep-80-965-s001.pdf]

SUPPLEMENTAL MATERIAL

Antagonistic effects of the cytotoxic molecules granzyme B and TRAIL in the immunopathogenesis of sclerosing cholangitis

Mareike Kellerer[1,2*], Sana Javed[1,2,3*], Christian Casar[2,4,5], Nico Will[2,4], Laura K. Berkhout[1,2], Dorothee Schwinge[2,4], Christian F. Krebs[2,6], Christoph Schramm[2,4,7], Katrin Neumann[1,2#], Gisa Tiegs[1,2#]

[1]Institute of Experimental Immunology and Hepatology, University Medical Centre Hamburg-Eppendorf, Hamburg, Germany

[2]Hamburg Center for Translational Immunology (HCTI), University Medical Centre Hamburg-Eppendorf, Hamburg, Germany

[3]Current address: Department of Pharmacy, The University of Faisalabad, Pakistan

[4]I. Department of Medicine, University Medical Center Hamburg-Eppendorf, Hamburg, Germany

[5]Bioinformatics Core, University Medical Centre Hamburg-Eppendorf, Hamburg, Germany

[6]III. Department of Medicine, University Medical Center Hamburg-Eppendorf, Hamburg, Germany

[7]Martin Zeitz Centre for Rare Diseases, University Medical Centre Hamburg-Eppendorf, Hamburg, Germany

[*]These authors share first authorship

[#]These authors share senior authorship

Table of content: Supporting Materials and Methods Section, 5 figures, 2 tables

SUPPORTING MATERIALS AND METHODS

Determination of liver injury. Heart blood was drawn from individual mice and liver injury was quantified by automated measurement of plasma activities of alanine transaminase (ALT) using a COBAS Mira System (Roche Diagnostic, Mannheim, Germany).

Hydroxyproline assay. The concentration of the collagen component hydroxyproline was determined in liver tissue by a spectrophotometric assay as previously described.[1]

Immunohistochemistry. Sirius red staining was done with 3 µm paraffin-embedded liver sections as previously described[2] to determine collagen fibers. To identify cholangiocytes, 3 µm paraffin-embedded liver sections were stained with an anti-CK19 antibody (TROMA-III; DSHB, Iowa City, IA, USA). A biotin-conjugated secondary antibody was used (Abcam, Cambridge, UK). For antigen detection, the DAB+ Substrate Chromogen System (Agilent Technologies, Santa Clara, CA, USA) was used. To stain myofibroblasts, 3 µm paraffin-embedded liver sections were incubated with an anti-αSMA antibody (1A4; Abcam, Cambridge, UK). After incubation with a biotin-conjugated secondary antibody (ImmunoReagents, Raleigh, NC, USA), liver sections were stained with the DAB+ Substrate Chromogen System. CK19 and αSMA staining was evaluated per μm^2 using the Keyence BZ-II Analyzer software.

Phospho-protein staining. Following extracellular staining, cells were fixed with 4% PFA. Cells were permeabilized with ice-cold methanol and stained with anti-phospho-Zap-70 (65E4, PE) and anti-phospho-PLCγ1 antibodies (D6M9S, Alexa Fluor 488; both Cell Signaling, Danvers, MA, USA).

Annexin V/7-AAD staining. To analyze apoptotic immune cells, cells were stained with Annexin V and 7-AAD using the PE Annexin V Apoptosis Detection Kit with 7-AAD (BioLegend) according to manufacturer's protocol.

Determination of GzmB in culture supernatants. To measure GzmB concentrations in supernatants of re-stimulated NPCs, an enzyme-linked immunosorbent assay (ELISA) was carried out according to manufacturer's protocol (R&D Systems, Minneapolis, MN). GzmB levels were measured using a Tecan Infinite plate reader (Tecan, Männedorf, Switzerland) and Magellan Data Analysis Software (V6.5; Tecan).

Quantitative real-time PCR analysis. Total RNA was isolated from shock-frozen liver tissue using the NucleoSpin RNA Midi Kit (Macherey-Nagel, Düren, Germany). Genomic DNA was digested using the DNA-*free* Kit (ThermoFisher Scientific). 1 µg RNA was transcribed into cDNA using the Verso cDNA Synthesis Kit (ThermoFisher Scientific) and the Biometra TAdvanced thermal cycler (Analytik Jena, Jena, Germany). Quantitative RT-PCR was performed using the PowerUp SYBR Green Master Mix (ThermoFisher Scientific) and the QuantStudio 7 Flex (Applied Biosystems, Waltham, USA). Relative mRNA levels were calculated using the ΔΔCT method after normalization to the reference gene β-actin. Quantification was shown in x-fold changes to the corresponding control cDNA. Primers were designed for detection of exon overlapping amplicons and were obtained from Metabion (Martinsried, Germany). Sequences of the primers are listed in Table S2.

Tables

Table S1: Antibodies used for cell surface staining.

Target	Fluorochrome	Clone	Distributor
CD4	BV 711	RM4-5	BioLegend
CD4	APC-Cy7	GK1.5	BioLegend
CD8	BV 785, BV 650	53-6.7	BioLegend
CD25	PE	PC-61	BioLegend
CD69	APC	H1.2F3	BioLegend
TCRβ	PE-Cy7	H57-597	BioLegend
TCRδ	PerCP-Cy5.5	GL3	BioLegend
CD1d-Tetramer	Alexa Fluor 647		NIH Tetramer Core Facility, Atlanta GA
NKp46	APC, BV 711	29A1.4	BioLegend
NK1.1	BV 605	PK136	BioLegend
CD107a	FITC	1D4B	BioLegend
TRAIL	PerCP-Cy5.5, PE	N2B2	BioLegend
Sca-1	BV 421	D7	BioLegend
KLRG1	BV 785	2F1/KLRG1	BioLegend
ICOS	FITC	7E.17G9	eBioscience
CD11c	FITC	N418	BioLegend
MHC-II	APC-Cy7	M5/114.15.2	BioLegend

Table S2: Sequences of the primer used for mRNA analysis.

Target	Forward primer<br>Reverse primer	Amplicon length	Annealing temperature
Timp1	5'-CGGACCTGGATGCTAAAAGGA-3'<br>3'-ACTCTCCAGTTTGCAAGGGA-5'	300 bp	60 °C
Col1a1	5'-GAGCGGAGAGTACTGGATCG-3'<br>3'-TACTCGAACGGGAATCCATC-5'	204 bp	60 °C
Col3a1	5'-GTCCACGAGGTGACAAAGGT-3'<br>3'-GATGCCCACTTGTTCCATCT-5'	204 bp	60 °C
Ccl2	5'-AGCTGTAGTTTTTGTCACCAAGC-3'<br>3'-GTGCTGAAGACCTTAGGGCA-5'	155 bp	60 °C
Cxcl2	5'-AGGCTACAGGGGCTGTTGT-3'<br>3'-TTCAGGGTCAAGGCAAACTT-5'	176 bp	60 °C
Cxcl1	5'-CCCAAACCGAAGTCATAGCCA-3'<br>3'-CTCCGTTACTTGGGGACACC-5'	118 bp	60 °C
Ccl4	5'-CTGTGCAAACCTAACCCCGA-3'<br>3'-AGGGTCAGAGCCCATTGGT-5'	118 bp	60 °C
IL-12	5'-AGACCCTGCCCATTGAACTG-3'<br>3'-GGCGGGTCTGGTTTGATGAT-5'	99 bp	60 °C

IFNγ	5'-ACAGCAAGGCGAAAAAGGATG-3'<br>3'-TCTTCCCCACCCCGAATCA-5'	171 bp	60 °C
Cxcl10	5'-GCCGTCATTTTCTGCCTCAT-3'<br>3'-TGCAGCGGACCGTCCTT-5'	76 bp	60 °C
Ccl28	5'-GCCTCACCTGAGTCATTGCC-3'<br>3'-CCATGGGAAGTATGGCTTCTGA-5'	121 bp	60 °C
Cxcl16	5'-TTGGACCCTTGTCTCTTGCG-3'<br>3'-CCAGTTCCACACTCTTTGCG-5'	292 bp	60 °C
Actb	5'-TATTGGCAACGAGCGGTTCC-3'<br>3'-GGCATAGAGGTCTTTACGGATGTC-5'	180 bp	60 °C

References

[1] Uchinami H, Seki E, Brenner DA, D'Armiento J. Loss of MMP 13 attenuates murine hepatic injury and fibrosis during cholestasis. Hepatology. 2006;44:420-9.

[2] Segnani C, Ippolito C, Antonioli L, Pellegrini C, Blandizzi C, Dolfi A, et al. Histochemical detection of collagen fibers by sirius red/fast green is more sensitive than van Gieson or sirius red alone in normal and inflamed rat colon. PLoS One. 2015;10:e0144630.

SUPPLEMENTAL FIGURE 1

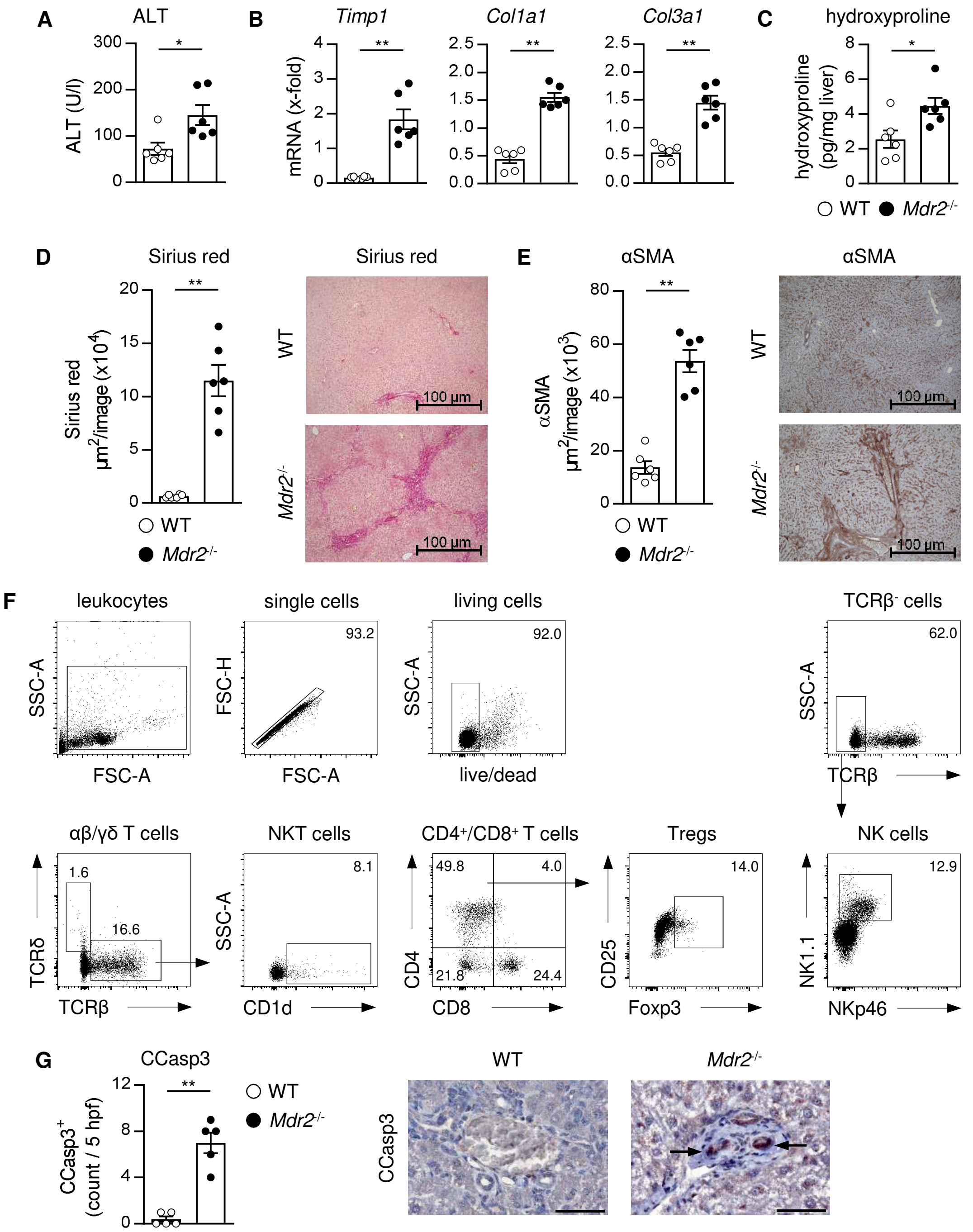

Figure S1. Disease pathology and leukocyte populations in *Mdr2*$^{-/-}$ mice. (A) Plasma ALT activities were determined in 12 weeks old *Mdr2*$^{-/-}$ and C57BL/6 WT mice. (B) Hepatic mRNA expression was analysed in *Mdr2*$^{-/-}$ mice and normalized to WT mice. (C) Hydroxyproline concentrations in liver tissue. (D) Sirius red and (E) αSMA stainings were quantified in liver sections. (F) Leukocytes were isolated from livers of 12 weeks old *Mdr2*$^{-/-}$ mice and stained for TCRβ, TCRδ, CD4, CD8, CD1d, NKp46 and NK1.1 to identify CD4^{+} TCRβ^{+} T cells, CD8^{+} TCRβ^{+} T cells, CD25^{+} Foxp3^{+} CD4^{+} Tregs, CD1d^{+} TCRβ^{+} NKT cells, NKp46^{+} NK1.1^{+} TCRβ^{-} NK cells and TCRδ^{+} γδ T cells. Representative frequencies and dot plots are shown. (G) CCasp3 was stained in liver sections and CCasp3-expressing cholangiocytes were counted. Arrows mark CCasp3^{+} cholangiocytes. Bars represent 50 µm. Mean ± SEM of one out of two experiments are shown. *p≤ 0.05, **p≤ 0.01.

SUPPLEMENTAL FIGURE 2

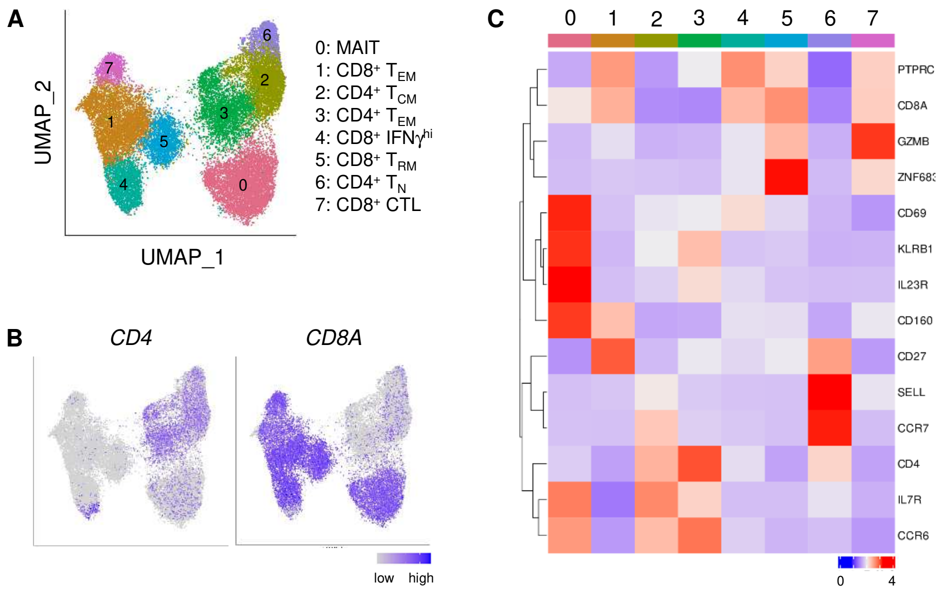

Figure S2. Hepatic T cell subsets in patients with PSC. (A) UMAP plot shows clustering of $CD3^{+}$ T cell subsets in livers of PSC patients undergoing liver transplantation. (B) UMAP plots depict $CD4^{+}$ and $CD8^{+}$ T cells clusters. (C) Heat map shows expression of subset-defining genes within the T cell clusters.

SUPPLEMENTAL FIGURE 3

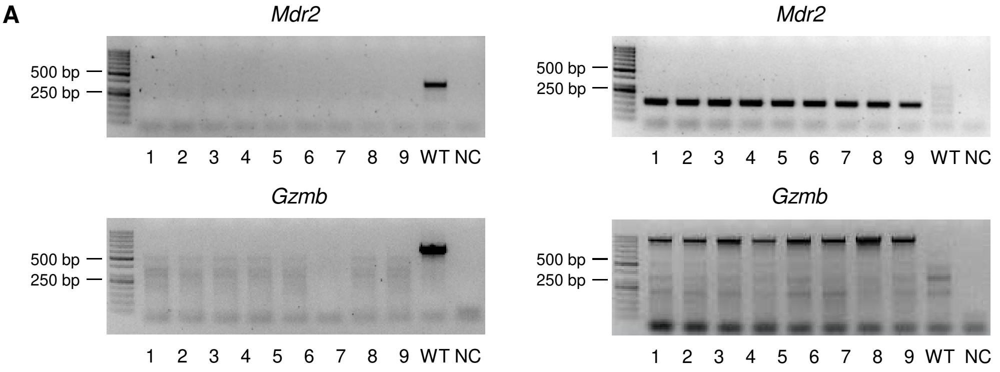

Gene	Sequence	PCR product
Mdr2	P1: CCACAGCCACACACTGACCT<br>P2: CATCAAACCACGTGCAGAAAA<br>P3: CCAGACTGCCTTGGGAAAAG	WT: 370 bp<br>(P1 + P2)<br>KO: 170 bp<br>(P1 + P3)
Gzmb	P1: ACACAAGTACTCAGAAGAGG<br>P2: TGAACACTGGGGAACCACT<br>P3. TGTGCTCGACGTTGTCACTG<br>P4: TGAACACTGGGGAACCACT	WT: 600 bp<br>(P1 + P2)<br>KO: 1000 bp<br>(P3 + P4)

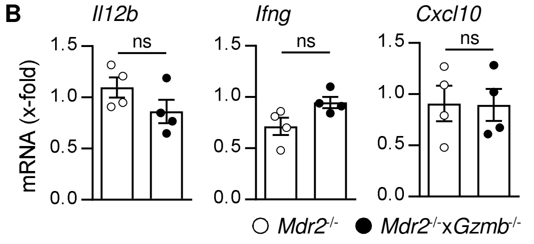

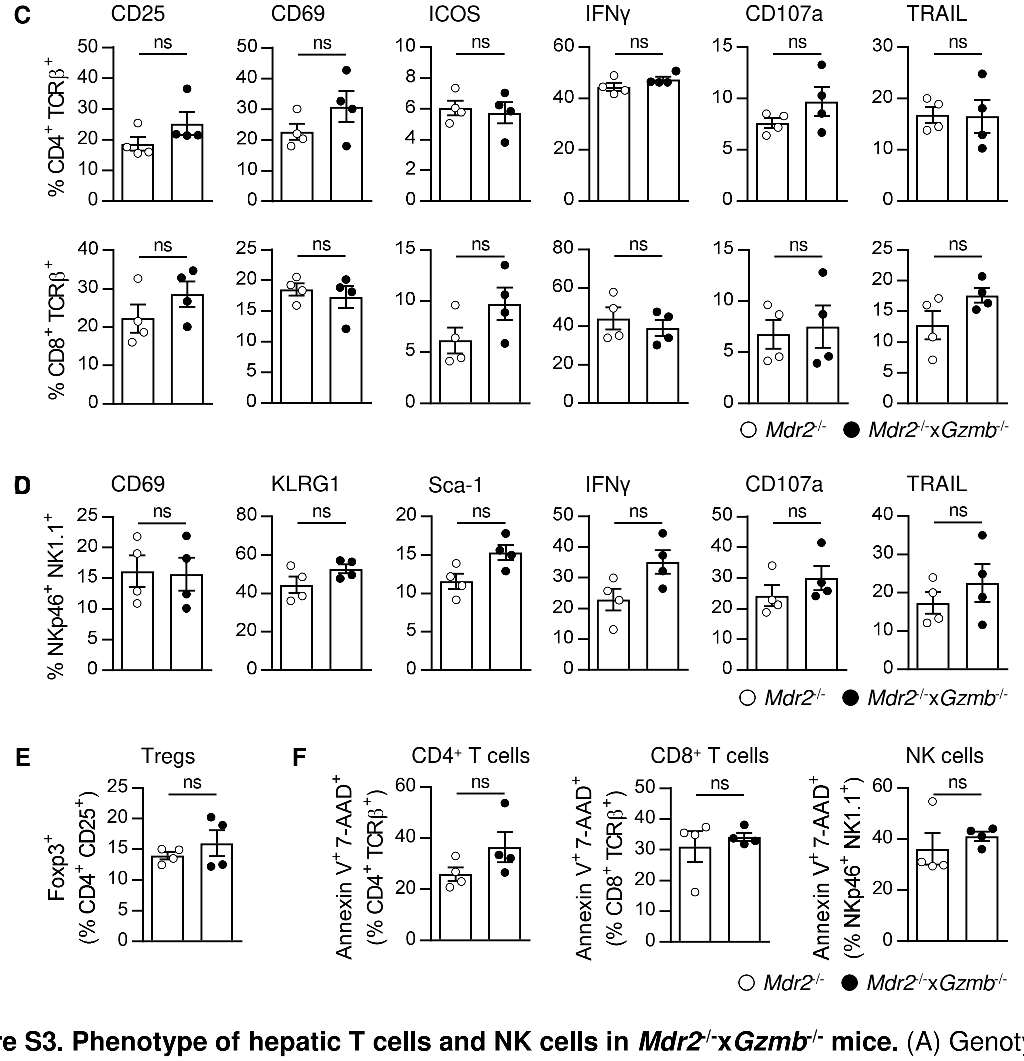

Figure S3. Phenotype of hepatic T cells and NK cells in *Mdr2*$^{-/-}$x*Gzmb*$^{-/-}$ mice. (A) Genotyping of *Mdr2*$^{-/-}$x*Gzmb*$^{-/-}$ mice is shown. (B) Hepatic mRNA expression was analysed in *Mdr2*$^{-/-}$x*Gzmb*$^{-/-}$ mice and normalised to *Mdr2*$^{-/-}$ mice. (C) Phenotype of hepatic T cells and (D) NK cells was analysed. (E) Frequencies of hepatic Tregs and (F) apoptotic T cells and NK cells. Mean ± SEM of one out of three experiments is shown. ns: not significant.

SUPPLEMENTAL FIGURE 4

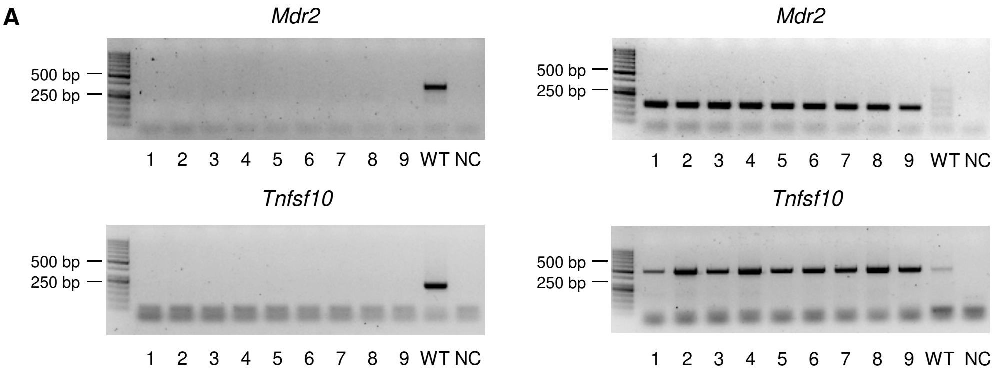

Gene	Sequence	PCR product
Mdr2	P1: CCACAGCCACACACTGACCT<br>P2: CATCAAACCACGTGCAGAAAA<br>P3: CCAGACTGCCTTGGGAAAAG	WT: 370 bp<br>(P1 + P2)<br>KO: 170 bp<br>(P1 + P3)
Tnfsf10	P1: AAAGACGGATGAGGATTTCTGGG<br>P2: GACAGAACACCATATTGCTGGCG<br>P3: GCCCTGAATGAACTGCAGGACG<br>P4: CACGGGTAGCCAACGCTATGTC	WT: 200 bp<br>(P1 + P2)<br>KO: 500 bp<br>(P3 + P4)

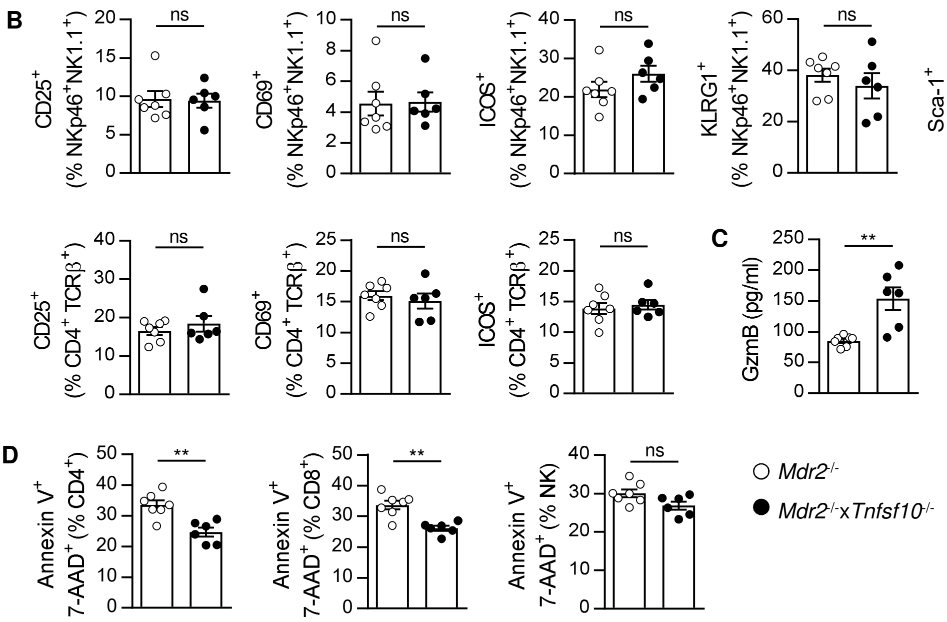

Figure S4. *Mdr2*$^{-/-}$x*Tnfsf10*$^{-/-}$ mice. (A) Genotyping of *Mdr2*$^{-/-}$x*Tnfsf10*$^{-/-}$ mice is shown. (B) Phenotype of hepatic NK cells and CD4^{+} T cells was analysed in 12 weeks old *Mdr2*$^{-/-}$x*Tnfsf10*$^{-/-}$ and *Mdr2*$^{-/-}$ mice. (C) GzmB levels were determined in culture supernatants of hepatic non-parenchymal cells from *Mdr2*$^{-/-}$x*Tnfsf10*$^{-/-}$ and *Mdr2*$^{-/-}$ mice. (D) Frequency of apoptotic hepatic CD4^{+} and CD8^{+} T cells and NK cells is shown. Mean ± SEM of one out of three experiments is shown. **p≤0.01 ; ns: not significant.

SUPPLEMENTAL FIGURE 5

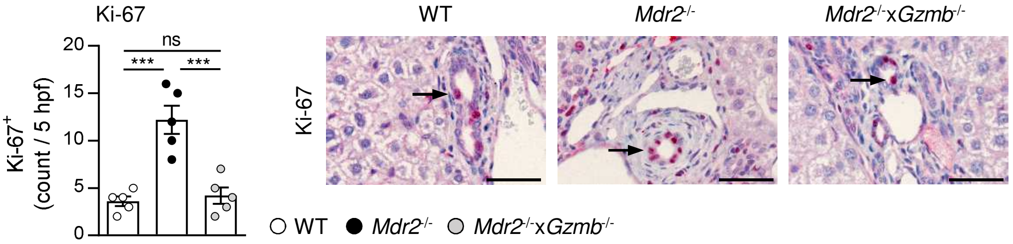

Figure S5. Proliferation of cholangiocytes in sclerosing cholangitis. Ki-67 was stained in liver sections of 12 weeks old WT, *Mdr2*$^{-/-}$ and *Mdr2*$^{-/-}$x*Gzmb*$^{-/-}$ mice. Ki-67-expressing cholangiocytes were counted. Bars represent 50 µm. Arrows mark Ki-67^{+} cholangiocytes. Mean ± SEM of one out of two experiments is shown.
